# Supplementary material for: Network structural dependency in the human connectome across the life-span
Source: Netw Neurosci. 2019 Jul 1;3(3):792–806. doi: 10.1162/netn_a_00081 (PMC6663353; doi:10.1162/netn_a_00081)
Supplement: Supplementary file 1 [file netn-03-792-s001.pdf]

Schirmer, M.D., Chung, A. W., Grant, P. E., & Rost, N. S. (2019). Supporting Information for “Network Structural Dependency in the Human Connectome Across the Life-Span.” *Network Neuroscience*, *3*(3), 792-806. https://doi.org/10.1162/netn_a_00081

Supplementary Materials

Network Structural Dependency in the Human Connectome Across the Life-Span

Markus D. Schirmer[a,b,c,1*], Ai Wern Chung[d*], P. Ellen Grant[d] and Natalia S. Rost[a]

[a]Stroke Division & Massachusetts General Hospital, J. Philip Kistler Stroke Research Center, Harvard Medical School, Boston, USA

[b]Computer Science and Artificial Intelligence Lab, Massachusetts Institute of Technology, Cambridge, USA

[c]Department of Population Health Sciences, German Centre for Neurodegenerative Diseases (DZNE), Germany

[d]Fetal-Neonatal Neuroimaging & Developmental Science Center, Boston Children’s Hospital, Harvard Medical School, Boston, MA, USA

*Authors contributed equally.

1 Corresponding author: mschirmer1@mgh.harvard.edu

Table S1: Number of unique nodes per anatomical label in the Craddock200 atlas.

Anatomical Label	Number of Nodes	
	Left Hemisphere	Right Hemisphere
Angular	1	1
Caudate	1	1
Cingulate anterior/posterior	1/2	2/2
Central Opercular	1	1
Frontal Medial	1	N/A
Frontal Orbital	2	1
Frontal Pole	10	10
Hippocampus	1	1
Heschl's	N/A	1
Insular	3	2
Intracalcarine	N/A	1
Inferior Frontal pars triangularis	1	1
Inferior Temporal posterior	2	2
Inferior Temporal temporooccipital	1	1
Juxtapositional Lobule	N/A	1
Lingual	2	2
Lateral Occipital inferior/superior	2/6	3/6
Middle Frontal	3	4
Middle Temporal anterior/posterior	1/2	1/1
Middle Temporal temporooccipital	1	2
Occipital Fusiform	2	1
Occipital Pole	4	3
Pallidum	1	1
Putamen	1	1

Precuneous	2	3
Paracingulate	2	2
Postcentral	4	4
Precentral	4	3
Parahippocampal posterior	1	1
Parietal Operculum	1	2
Planum Temporale	2	N/A
Planum Polare	N/A	1
Subcallosal	1	N/A
Superior Frontal	3	2
Supramarginal anterior/posterior	1/1	1/1
Superior Parietal Lobule	2	2
Superior Temporal posterior	N/A	2
Thalamus	2	2
Temporal Fusiform anterior/posterior	1/1	1/2
Temporal Occipital Fusiform	2	1
Temporal Pole	3	3
**Total number of nodes**	84	86

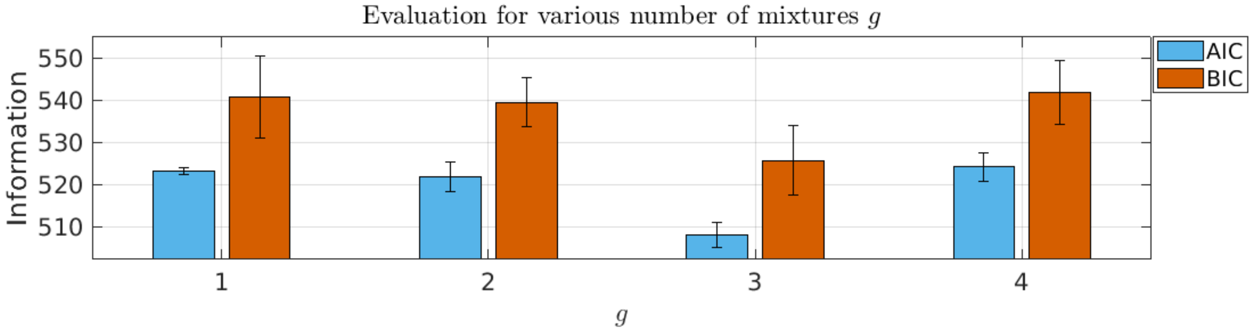

Figure S1: GMM assessment for varying number of Gaussians g fitted to each of the four group connectomes, based on Akaike Information Criterion (AIC) and Bayesian Information Criterion (BIC). Both measure suggest that g=3 best describes the observed data.

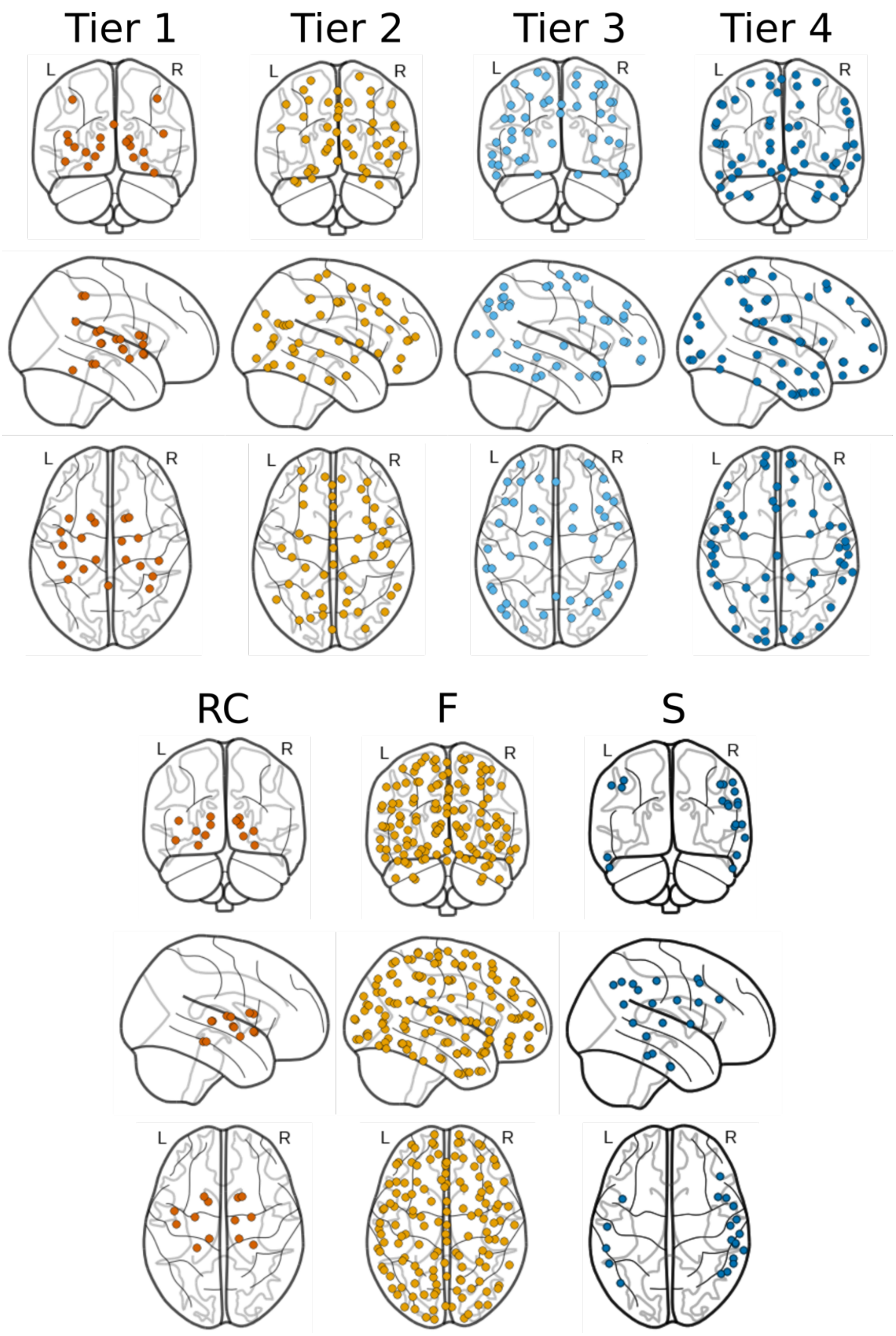

Figure S2: Brain regions plotted by subnetwork label for each framework. Top: NDI framework with four Tiers (decreasing importance). Bottom: RC framework with rich-club (RC), Feeder (F), and Seeder (S) subnetworks.

a)

b)

c)

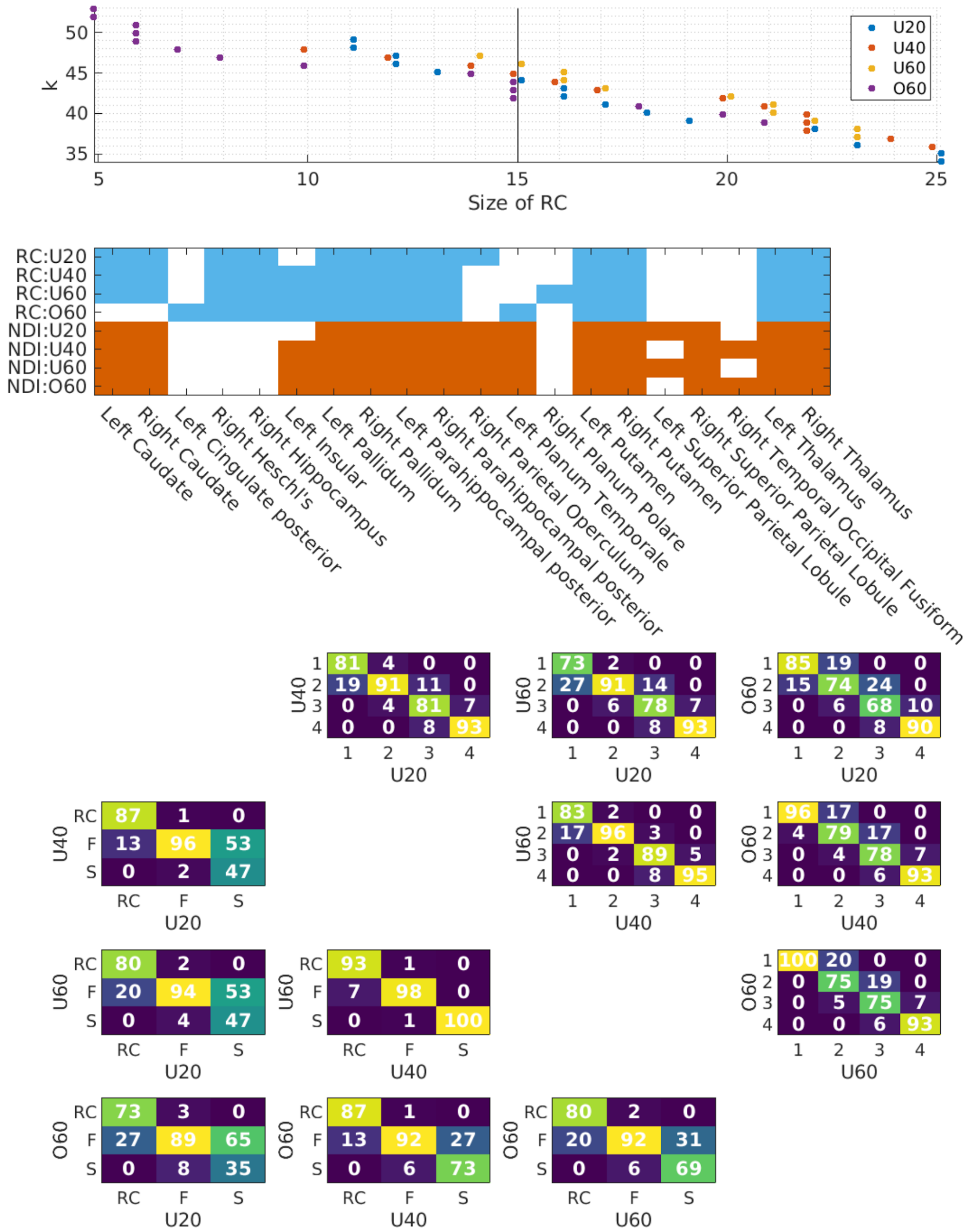

Figure S3: Analysis with consistent number of rich-club nodes between age groups, but variable choice of k (44, 45, 46, and 44 for U20, U40, U60, and O60, respectively). a) Analysis of size of rich-club with varying degree k for each age group. The graph demonstrates that consistent rich-club size analysis is only possible for 15 nodes in this data set. b) Rich-club and the top 15

brain regions with highest NDI scores. c) Percentage of nodal assignment to subnetworks/Tiers between groups. The upper right corresponds to comparisons using the NDI framework, whereas the lower left utilizes the RC framework with 15 nodes.
